# The Intricacies of Sprott-B System with Fractional-Order Derivatives: Dynamical Analysis, Synchronization, and Circuit Implementation

**DOI:** 10.3390/e25091352

**Published:** 2023-09-17

**Authors:** Rending Lu, Prasina Alexander, Hayder Natiq, Anitha Karthikeyan, Sajad Jafari, Jiri Petrzela

**Affiliations:** 1 School of Electronic Engineering, Changzhou College of Information Technology, Changzhou 213164, China; 13584580128@163.com; 2 Centre for Nonlinear Systems, Chennai Institute of Technology, Chennai 600069, India; prasinaa@citchennai.net; 3 Department of Computer Technology Engineering, College of Information Technology, Imam Ja’afar Al-Sadiq University, Baghdad 10001, Iraq; haydernatiq86@gmail.com; 4 Department of Electronics and Communication Engineering, Vemu Institute of Technology, Chithoor 517112, India; mrs.anithakarthikeyan@gmail.com; 5 Department of Electronics and Communications Engineering, University Centre for Research & Development, Chandigarh University, Mohali 140413, India; 6 Health Technology Research Institute, Amirkabir University of Technology (Tehran Polytechnic), Tehran 15916-34311, Iran; sajadjafari83@gmail.com; 7 Department of Biomedical Engineering, Amirkabir University of Technology (Tehran Polytechnic), Tehran 15916-34311, Iran; 8 Department of Radio Electronics, Brno University of Technology, 616 00 Brno, Czech Republic

**Keywords:** Sprott-B system, fractional order, dynamical analysis, circuit implementation

## Abstract

Studying simple chaotic systems with fractional-order derivatives improves modeling accuracy, increases complexity, and enhances control capabilities and robustness against noise. This paper investigates the dynamics of the simple Sprott-B chaotic system using fractional-order derivatives. This study involves a comprehensive dynamical analysis conducted through bifurcation diagrams, revealing the presence of coexisting attractors. Additionally, the synchronization behavior of the system is examined for various derivative orders. Finally, the integer-order and fractional-order electronic circuits are implemented to validate the theoretical findings. This research contributes to a deeper understanding of the Sprott-B system and its fractional-order dynamics, with potential applications in diverse fields such as chaos-based secure communications and nonlinear control systems.

## 1. Introduction

Chaotic systems are of great importance in various fields [1]. Due to the chaotic systems’ sensitivity to initial conditions, researchers can better understand the dynamics of particular phenomena, such as weather patterns and population dynamics [2,3,4]. Additionally, chaotic systems include underlying structures and patterns that make them useful for prediction applications like stock market research [5,6]. Cryptography is another significant application of chaotic systems, which uses encryption methods and secure data transmission [7,8,9]. Encrypted data cannot be decoded by unauthorized parties without knowledge of the system’s initial conditions or parameters [10,11]. Simple chaotic systems can provide a simplified framework and can be an efficient tool for the mentioned applications [12,13,14,15]. The chaotic systems are also useful in nonlinear programming (optimization) [16].

Recently, fractional calculus has drawn a lot of attention [17,18]. In comparison to integer-order derivatives, fractional-order derivatives offer a more accurate description of events that occur in reality [19]. Fractional calculus makes it possible to express the non-integer dynamics of many physical systems in more detail. Fractional-order derivatives add a further degree of freedom, making the system dynamics more complex [20]. Richer and more elegant chaotic behavior could arise from this complexity, improving researchers’ understanding of the underlying causes [21,22]. Due to more parameters of fractional derivatives, they can improve chaotic systems’ potential for synchronization and control applications [23,24,25]. In addition, studies have demonstrated that because of their greater complexity, fractional-order chaotic systems are more robust against noise and offer stronger security standards and more resistance to attacks in encryption schemes [24,26]. As a result, numerous researchers have attempted to use fractional-order derivatives to study the dynamics of chaotic systems and maps [27,28,29]. The efficiency of the fractional-order system in applications such as modeling, image encryption, and filtering has been previously explored [30,31,32].

In practical applications, electronic circuits of chaotic systems are required [33,34]. Chaotic circuits simplify the investigation and comprehension of chaotic behaviors by offering a physical representation of nonlinear dynamical systems [26,35,36]. Incorporating components such as transistors or amplifiers can induce nonlinearities into the circuit, leading to chaos [37]. The analog circuit implementation of fractional-order systems can be challenging due to the non-integer nature of the system’s order [38]. There are several methods for implementing circuits for fractional-order systems. Linear approximation is a common method for studying fractional-order systems [39]. In this method, frequency domain techniques based on Bode diagrams are employed. Then, a corresponding electronic circuit can be designed using passive components such as resistors and capacitors. The circuits of fractional-order systems have been proposed in several studies.

Motivated by the above discussion in this paper, a simple chaotic system, Sprott-B, is analyzed through fractional derivatives. Previous studies on the Sprott-B system have mainly focused on its qualitative behavior and bifurcation analysis using integer-order derivatives. However, a comprehensive dynamical analysis involving bifurcation diagrams and coexisting attractors has not been conducted for this system with fractional-order derivatives. This research fills this gap by providing a detailed analysis of the system’s dynamics using fractional-order derivatives. Moreover, there is limited knowledge about how the synchronization properties of the Sprott-B system change when fractional-order derivatives are introduced. This research investigates the synchronization behavior for different derivative orders. Therefore, studying the system’s behavior with fractional derivatives in detail contributes to a deeper understanding of its complex dynamics, representing coexisting attractors. Finally, this research validates the numerical findings by implementing integer-order and fractional-order electronic circuits that replicate the dynamics of the Sprott-B system. Designing circuits with non-integer orders and that maintain stability and accuracy is a significant challenge. In the next section, the model is introduced. In Section 3, the system’s equilibrium point and dynamics are explored through bifurcation diagrams. The coexisting attractors and their basins are also shown in different derivatives. Then, the synchronized behavior of fractional-order Sprott-B is investigated. Finally, the circuits of the integer-order and fractional-order systems are implemented. The conclusion of this paper is provided in Section 4.

## 2. Model

In 1994, Sprott introduced some simple chaotic systems [40]. One of these systems is the Sprott-B system, defined as follows:

(1) $$\begin{array}{l} \dot{x}=yz,\\ \dot{y}=x-y,\\ \dot{z}=a-xy. \end{array}$$

The fractional form of this system can be written as follows:

(2) $$\begin{array}{l} D^{q}x=yz,\\ D^{q}y=x-y,\\ D^{q}z=a-xy, \end{array}$$

where $D^{q}$ shows the Caputo-type fractional derivative of order q. The Caputo-type derivative is defined as

(3) $$D^{q}x_{t}=\frac{1}{\Gamma \left(\lceil q\rceil -q\right)}\int_{0}^{t}{\left(t-\tau \right)}^{\lceil q\rceil -q-1}x_{t}^{\left(\lceil q\rceil \right)}d\tau$$

Applying the Riemann–Liouville integral operator (J) [41] on this equation leads to

(4) $$D^{q}x_{t}=J^{\left(\lceil q\rceil -q\right)}\frac{d^{\left\lceil q\right\rceil }x_{t}}{dt^{\left\lceil q\right\rceil }}.$$

Thus, it can be solved numerically by using the Adam–Bashforth–Moulton algorithm [41].

## 3. Results

### 3.1. Stability Analysis

**Figure 1:**
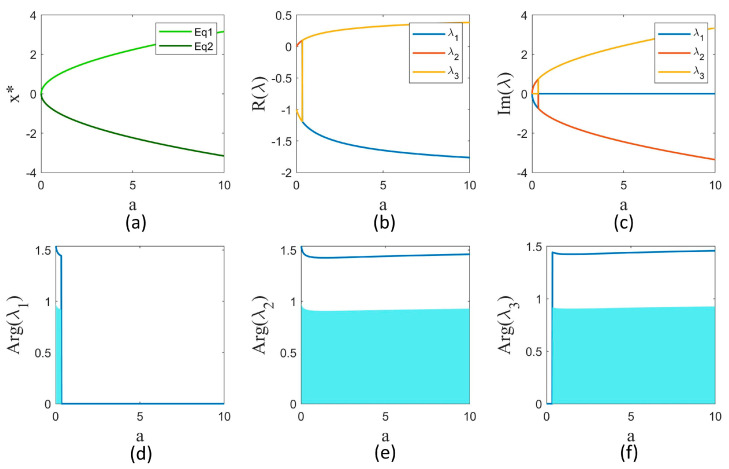
(**a**) The equilibrium points ($x^{*}$) of system (2) as a function of parameter a. (**b**,**c**) The real and imaginary parts of the eigenvalues of the Jacobian of the system at $x^{*}$ as a function of parameter a. (**d**–**f**) The argument of the eigenvalues as a function of parameter a. The blue region represents the area for the fractional order q for which that eigenvalue meets condition (8).

The equilibria ($X^{*}$) of the system $D^{q}X=F\left(X\right)$ can be found by F(X)=0. Therefore, for system (2), one can write

(5) $$\begin{array}{l} yz=0,\\ x-y=0,\\ a-xy=0, \end{array}$$

which results in $X^{*}=\pm \left(\sqrt{a},\sqrt{a},0\right)$. The equilibrium points according to parameter a are shown in Figure 1a. To analyze the stability of the equilibrium points, the Jacobian of system (2) should be found, which is as follows:

(6) $$J=\left[\begin{array}{ccc} 0 & z & y\\ 1 & -1 & 0\\ -y & -x & 0 \end{array}\right].$$

The characteristic equation of the Jacobian with the substitution of $X^{*}$ is as follows:

(7) $$\lambda^{3}+\lambda^{2}+a\lambda +2a=0.$$

This leads to three eigenvalues, whose real and imaginary parts are shown in Figure 1b,c according to parameter *a*. For the fractional-order systems, the equilibrium point is asymptotically stable if and only if

(8) |arg(λ)|>qπ/2.

where arg(λ) denotes the principal argument of λ. Figure 1d–f represent the argument of three eigenvalues for different parameter a values. For each eigenvalue, if the fractional order is located in the blue area, condition (8) holds for that eigenvalue. However, for any parameter a, all three eigenvalues must meet (8). According to the argument figures, the intersection of blue areas is null. Hence, the equilibrium is unstable for any parameter a and fractional order q.

### 3.2. Dynamical Analysis

**Figure 2:**
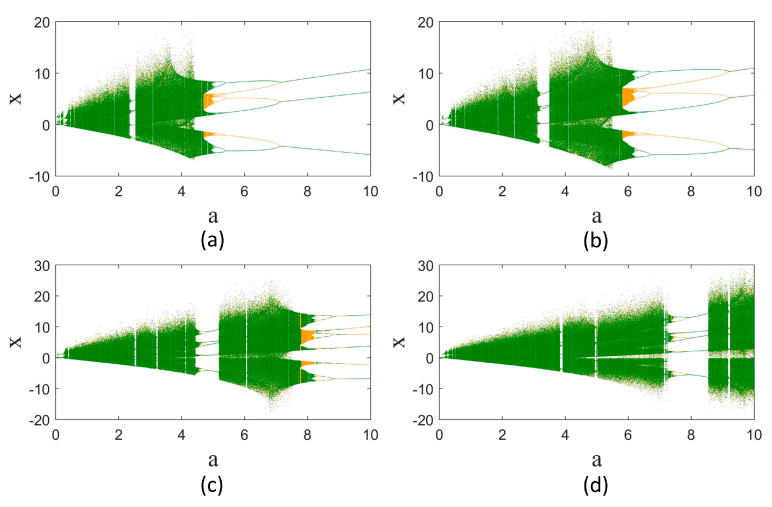
Bifurcation diagrams of the Sprott-B system according to parameter a in different derivative orders. (**a**) q=1, (**b**) q=0.98, (**c**) q=0.96, and (**d**) q=0.94. The green and yellow colors refer to the initial conditions [1 1 1] and [−1−1 1], respectively.

The model dynamics are considerably impressed by changing the derivative order to fractional. To represent this, the bifurcation diagrams of the model according to parameter a are obtained for different orders q. The result is shown in Figure 2. The bifurcation diagram of the original model (q=1) is illustrated in Figure 2a. The Sprott-B system has symmetry around the x and y variables. For some values of parameter *a*, the system’s attractor is symmetric, and for some values, there are two symmetric coexisting attractors. The bifurcation diagram for the symmetric coexisting attractors of the system is shown with green and yellow colors for initial conditions [1 1 1] and [−1−1 1]. The bifurcation diagrams for fractional orders q=0.98, 0.96, and 0.94 are shown in Figure 2b,c. It is observed that the region of chaotic dynamics is extended when considering the fractional-order derivatives. Moreover, the largest periodic window, which is [2.38 2.525] in q=1, enlarges and moves to larger a values by decreasing the derivative order. The largest periodic window for q=0.98 is within [3.2 3.48], that for q=0.96 is within [4.57 5.18], and that for q=0.94 is within [7.44 8.55].

**Figure 3:**
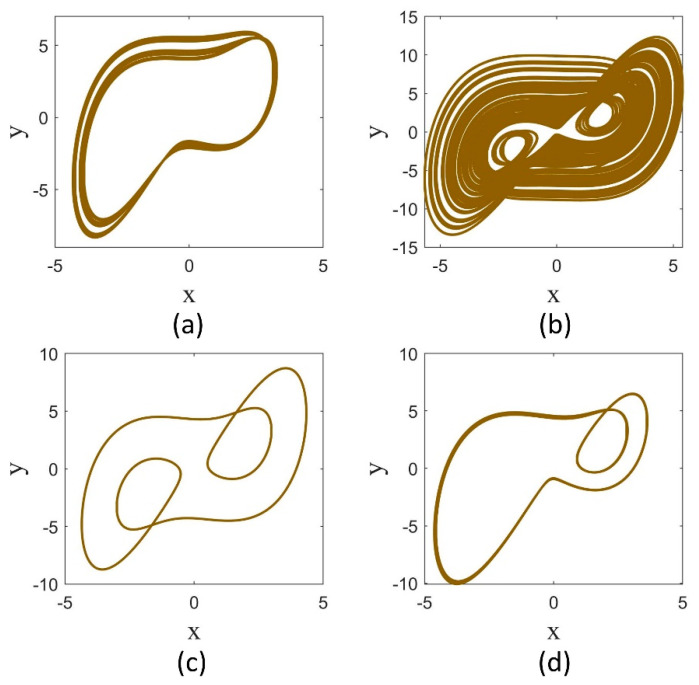
The attractors of the Sprott-B system in  a=5 for different derivative orders. (**a**) q=1, (**b**) q=0.98, (**c**) q=0.96, and (**d**) q=0.94.

As observed in the bifurcation diagrams for any a value, the dynamics are dependent on the fractional order. In other words, for a fixed a value, varying the fractional order leads the attractor to change to different chaotic or periodic ones. For example, the attractors of the system for q=1, 0.98, 0.96, 0.94 are shown in Figure 3. It can be seen that while the attractor for q=1 is a non-symmetric chaotic attractor, it is symmetric and chaotic for q=0.98, it is symmetric and periodic for q=0.96, and it is non-symmetric and periodic for q=0.94. The symmetric attractors remain the same under the transformation (x,y)→(−x,−y).

**Figure 4:**
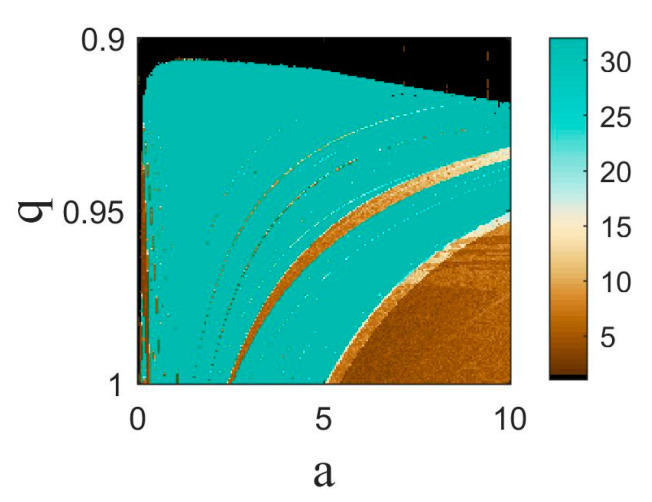
Two-dimensional bifurcation diagram of the Sprott-B system according to parameter a and fractional order q. The period of oscillations is shown by color.

In order to represent the simultaneous effect of parameter a and fractional order q on the system’s dynamics, the 2D bifurcation diagram is plotted in Figure 4. In this figure, the period of oscillations of the system is shown by color. The blue region represents the chaos region, the brown tone represents the periodic region, and the black area shows an oscillation death state. It is evident that as the fractional order decreases from q=1, the chaotic region is extended, and the periodic region starts in a larger a value. As q decreases from q=0.918, the oscillation death state is formed. For q<0.9055, the oscillations disappear completely.

**Figure 5:**
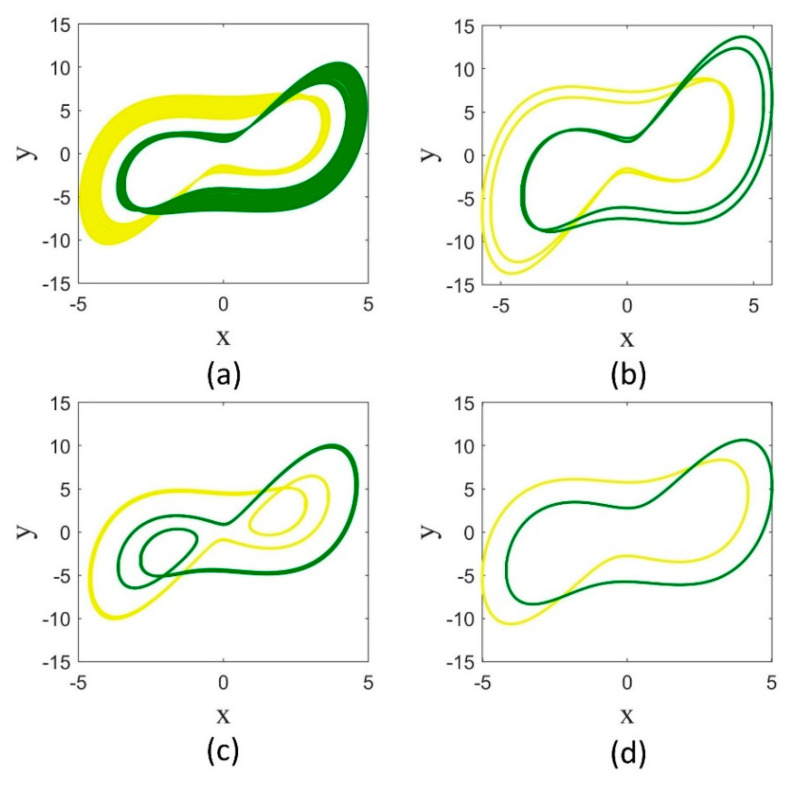
Coexisting attractors of the fractional-order Sprott-B system. (**a**) q=0.98, a=6; (**b**) a=0.96, a=6.5; (**c**) q=0.94, a=5; (**d**) q=0.98, a=8. The green and yellow colors refer to the initial conditions [1 1 1] and [−1−1 1], respectively.

**Figure 6:**
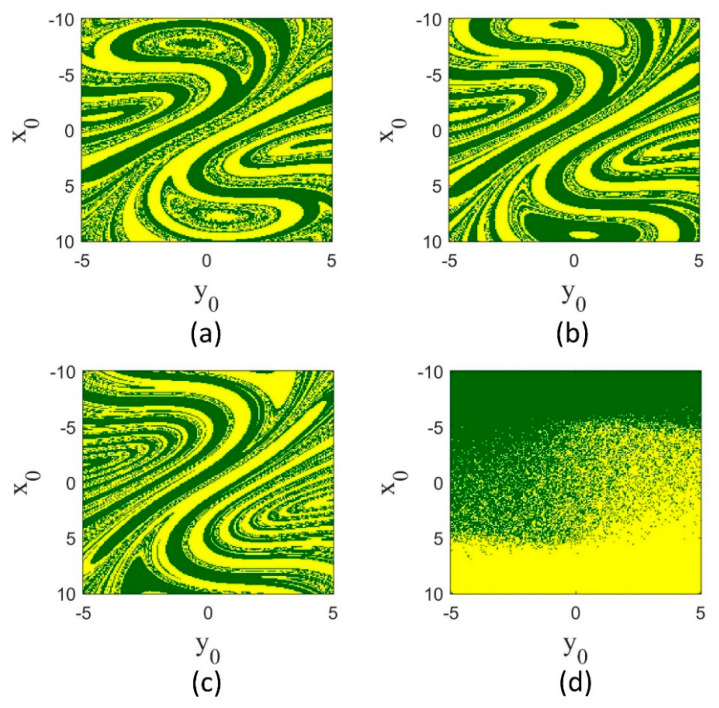
Basins of attraction of coexisting attractors of Sprott-B system for different derivative orders. (**a**) q=1, a=5; (**b**) q=0.98, a=6; (**c**) q=0.96, a=8; and (**d**) q=0.94, a=7.4. The green and yellow colors refer to two coexisting attractors.

Another remarkable point in the bifurcation diagrams is the bistability region. The range of parameter a in which coexisting attractors are formed differs by varying the fractional orders. Furthermore, a variety of periodic and chaotic coexisting attractors are generated. Some of the coexisting attractors for different a and q are demonstrated in Figure 5. In addition, the basin of attraction of coexisting attractors differs in different derivative orders. The basin of attraction for q=1 and a=5 is shown in Figure 6a, and that for q=0.98 and a=6 is shown in Figure 6b. These two basins have a similar pattern, although they are not the same. The basins for q=0.96 (a=8) and q=0.94 (a=7.4) are indicated in Figure 6c,d, respectively. In Figure 6c, the basins of two attractors are interwoven, similar to q=0.98 and q=1. However, in Figure 6d, the basins of two attractors are almost separated from each other.

### 3.3. Synchronization

In this subsection, we examine the synchronization ability of two fractional-order systems, which can be described as

(9) $$\begin{array}{c} \{\begin{array}{c} D^{q}x_{1}=y_{1}z_{1}+\sigma \left(x_{2}-x_{1}\right)\;\\ D^{q}y_{1}=x_{1}-y_{1}\;\;\;\;\;\;\;\;\;\\ D^{q}z_{1}=a-x_{1}y_{1}\;\;\;\;\;\;\;\; \end{array}\\ \{\begin{array}{c} D^{q}x_{2}=y_{2}z_{2}+\sigma \left(x_{1}-x_{2}\right)\\ D^{q}y_{2}=x_{2}-y_{2}\;\;\;\;\;\;\;\;\;\\ D^{q}z_{2}=a-x_{2}y_{2}\;\;\;\;\;\;\;\; \end{array} \end{array}$$

where σ represents the coupling coefficient. To measure the synchronization, the numerical error is computed for different a and by varying the coupling coefficient as follows:

(10) $$E=<\sqrt{{\left(x_{1}-x_{2}\right)}^{2}}{>}_{t},$$

where $<.{>}_{t}$ denotes the average over time.

**Figure 7:**
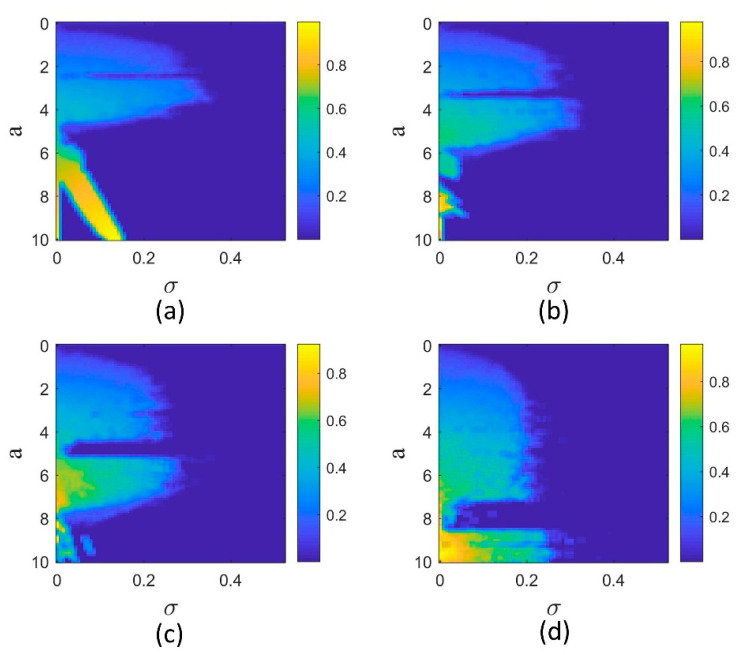
Normalized synchronization error of two coupled Sprott-B systems in 2D parameter plane (σ,a) for different derivative orders. (**a**) q=1, (**b**) q=0.98, (**c**) q=0.96, and (**d**) q=0.94. The synchronization region is shown by dark blue color.

The normalized errors in the 2D plane (a, σ) for orders q=1, 0.98, 0.96, and 0.94 are shown in Figure 7. It can be seen that the synchronization region (dark blue) shrinks as the fractional order decreases. The reason is that for q=1, the dynamics change to periodic in smaller a values, and the periodic systems need a lower coupling coefficient to synchronize. For lower fractional orders, the dynamics are mostly chaotic, and hence stronger coupling is required.

### 3.4. Circuit Implementation

**Figure 8:**
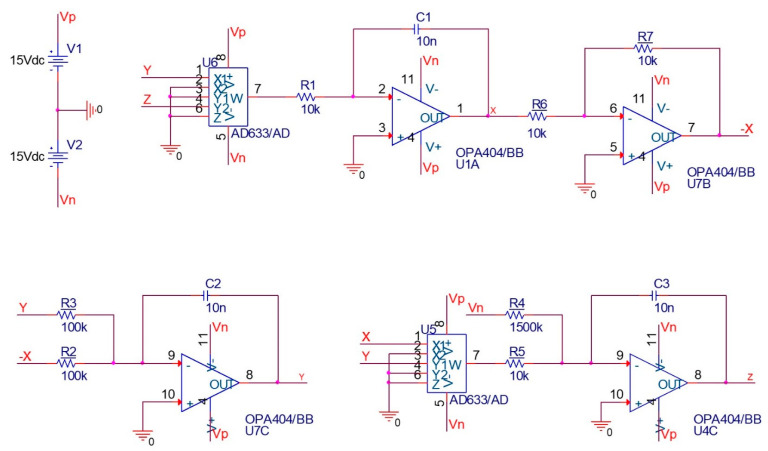
Schematic of the electronic circuit of inter-order Sprott-B system for a=1.

**Figure 9:**
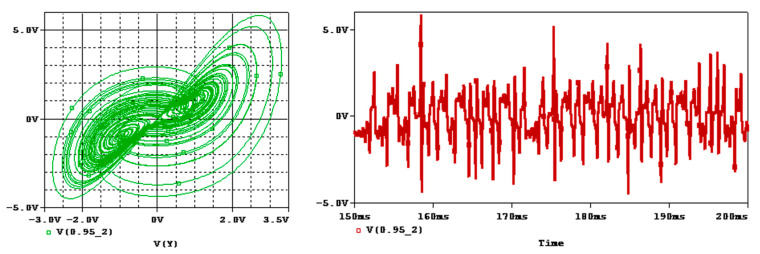
Attractor and time series of integer-order Sprott-B system for a=1 in OrCAD-PSpice.

The implementation of fractional-order chaotic systems is of particular importance in practical applications. In this subsection, firstly, the electronic circuit of the integer-order Sprott-B system is implemented in the OrCAD-PSpice environment. Then, the design of the fractional-order circuit is explained. Common electronic components such as the resistor, capacitor, and OPAMP are used, and AD633 is used as a multiplier. The system equations using electronic components can be written as follows:

(11) $$\begin{array}{l} \dot{x}=\frac{yz}{R_{1}C_{1}}\\ \dot{y}=\frac{x}{R_{2}C_{2}}-\frac{y}{R_{3}C_{2}}\\ \dot{z}=\frac{-V_{n}}{R_{4}C_{3}}-\frac{xy}{R_{5}C_{3}} \end{array}$$

where the parameter a of the system can be adjusted by choosing the proper $R_{4}$. The circuit elements can be chosen as $C_{1}=C_{2}=C_{3}=1\;\mathrm{nF}$, $R_{1}=R_{5}=10\;k$, $R_{2}=R_{3}=100\;k$, and $R_{4}=1500$ for a=1. Also, Vp=−Vn=15 v is chosen. The schematic of the integer-order circuit is shown in Figure 8. The time series and attractor of the system by setting zero initial conditions for the capacitors are shown in Figure 9, which match with the simulation results.

For the fractional-order system, we use the chain type fractance, which is constructed of a series of RC circuits. As mentioned in the introduction, linear approximations can be employed to analyze fractional-order integrators based on the frequency domain techniques applied to Bode diagrams. The transfer function of the chain fractance in the Laplace domain is as follows:

(12) $$H^{RC}\left(s\right)=\frac{1}{C_{1}s+\frac{1}{R_{1}}}+\ldots +\frac{1}{C_{N}s+\frac{1}{R_{N}}}$$

and the transfer function of an integrator of the order q is as follows:

(13) $$F\left(s\right)=\frac{1}{s^{q}}$$

The order of the approximation depends on the desired bandwidth and the difference between the actual and approximate magnitude Bode diagrams. Our aim is to validate the numerical simulation results using a circuit; hence, a third-order approximation seems sufficient. However, the more appropriate approximations can also be used for generating a wideband operational regime [42]. In the following, we present the circuit for *q* = 0.95; the circuit for other fractional orders can be obtained using a similar procedure.

For q=0.95 with the third-order approximation, we can write [43]

(14) $$\frac{1}{s^{0.95}}\approx \frac{1.2862s^{2}+18.6004s+2.0833}{s^{3}+18.4738s^{2}+2.6547s+0.003}$$

**Figure 10:**
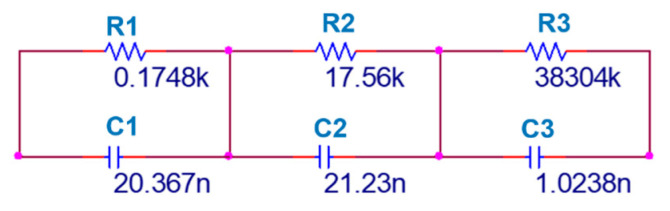
Schematic of the electronic circuit of the fractional integrator with q=0.95.

**Figure 11:**
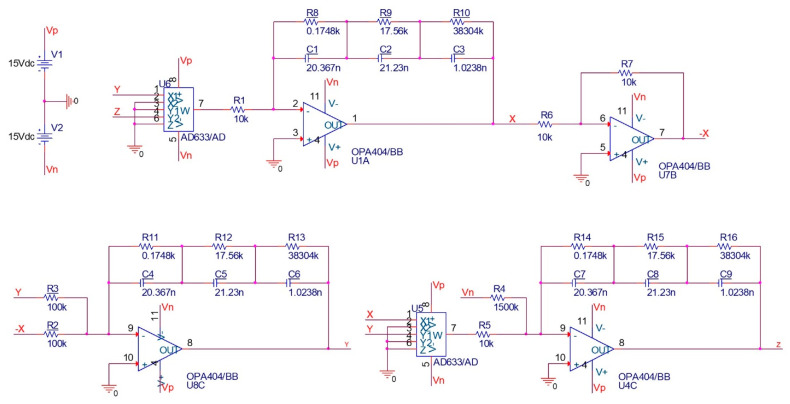
Schematic of the electronic circuit of fractional-order Sprott-B system for q=0.95 and a=1.

**Figure 12:**
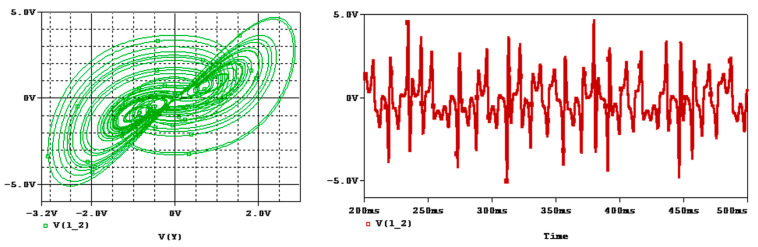
Attractor and time series of fractional-order Sprott-B system for q=0.95 and a=1 in OrCAD-PSpice.

Hence, the resistors and the capacitors can be chosen as $R_{1}=0.1748\;k\Omega$, $R_{2}=17.56\;k\Omega$, $R_{3}=38.304\;M\Omega$, $C_{1}=20.367\;\mathrm{nF}$, $C_{2}=21.23\;\mathrm{nF}$, and $C_{3}=1.0238\;\mathrm{nF}$. The circuit schematic of the fractional-order integrator and the fractional-order Sprott-B system are shown in Figure 10 and Figure 11, respectively. The results of simulating this circuit are shown in Figure 12. It can be observed that the chaotic attractor is well reproduced in the fractional-order circuit.

## 4. Conclusions

This paper studied the dynamics of a simple chaotic system known as the Sprott-B system with fractional-order derivatives. The system’s dynamics were investigated through bifurcation diagrams as a function of the system parameter for different fractional orders. It was shown that decreasing the derivative order from integer one extended the chaotic region, and the periodic behavior initiated from larger parameters. The fractional-order derivative also led to various coexisting attractors, the basins of which differed in different orders. In addition, it was shown that the synchronization of coupled Sprott-B systems depended on the fractional order. Analysis showed that the integer-order systems had a larger synchronization region, and decreasing the order shrunk this region. Finally, the electronic circuits of the integer-order and fractional-order systems were implemented using common components. The fractional integrator was implemented using chain-type fractance. The response of the circuits was in good agreement with the simulation results.

In the end, we provide some suggestions for future works. Future studies can consider conducting a stability analysis of the fractional-order Sprott-B system to determine its robustness against perturbations and noise. Moreover, exploring different synchronization schemes such as complete synchronization, generalized synchronization, or adaptive synchronization for various derivative orders can be considered. The enhancement of the existing fractional-order electronic circuit implementations can be planned to improve accuracy, reduce noise interference, and increase control capabilities.

## Data Availability

Not applicable.
